# Polymorphisms in *GP6*, *PEAR1A*, *MRVI1*, *PIK3CG*, *JMJD1C*, and *SHH* Genes in Patients with Unstable Angina

**DOI:** 10.3390/ijerph17207506

**Published:** 2020-10-15

**Authors:** Rafał Rudzik, Violetta Dziedziejko, Monika Ewa Rać, Marek Sawczuk, Agnieszka Maciejewska-Skrendo, Krzysztof Safranow, Andrzej Pawlik

**Affiliations:** 1 Department of Physiology, Pomeranian Medical University, 70-111 Szczecin, Poland; drrudzik@gmail.com; 2 Department of Biochemistry and Medical Chemistry, Pomeranian Medical University, 70-111 Szczecin, Poland; viola@pum.edu.pl (V.D.); carmon12@gmail.com (M.E.R.); chrissaf@mp.pl (K.S.); 3 Insitute of Physical Culture Sciences, University of Szczecin, 70-111 Szczecin, Poland; sawczuk_marek@wp.pl; 4 Faculty of Physical Culture, Gdansk University of Physical Education and Sport, 80-336 Gdansk, Poland; maciejewska.us@wp.pl

**Keywords:** coronary artery disease, unstable angina, venous thromboembolism, platelet aggregation, polymorphism

## Abstract

Introduction: Coronary artery disease (CAD) is a significant public health problem because it is one of the major causes of death worldwide. Several studies have investigated the associations between CAD and polymorphisms in genes connected with platelet aggregation and the risk of venous thromboembolism. Aim: In this study, we examined the associations between polymorphisms in *GP6* (rs1671152), *PEAR1A* (rs12566888), *MRVI1* (rs7940646), *PIK3CG* (rs342286), *JMJD1C* (rs10761741), *SHH* (rs2363910), and CAD in the form of unstable angina as well as selected clinical and biochemical parameters. The study enrolled 246 patients with diagnosed unstable angina and 189 healthy controls. Results: There were no significant differences in the distribution of the studied polymorphisms between the patients with unstable angina and the controls. In patients with the *GP6* rs1671152 GG genotype, we observed increased BMI values and an increased frequency of type 2 diabetes diagnosis. Conclusions: The results of this study suggest a lack of association between *GP6* (rs1671152), *PEAR1A* (rs12566888), *MRVI1* (rs7940646), *PIK3CG* (rs342286), *JMJD1C* (rs10761741), *SHH* (rs2363910), and unstable angina. The results indicate an association between *GP6* (rs1671152) and type 2 diabetes.

## 1. Introduction

Diseases of the circulatory system, including coronary artery disease (CAD), are important public health problems because they are major causes of death worldwide. Several studies have investigated the associations between CAD and polymorphisms in genes connected with platelet aggregation and the risk of venous thromboembolism. A genome-wide association study of European and American populations identified seven loci which may be associated with platelet aggregation: *JMJD1C*, *GP6*, *ADRA2A*, *PEAR1*, *SHH*, *PIK3CG*, and *MRVI1* [1]. However, the results are still controversial. CAD is a multifactorial disease that results from complex interactions between many genetic and environmental factors. Imbalance between platelets and the endothelium can cause the development of atherosclerotic lesions and cardiovascular diseases.

Glycoprotein VI (GPVI or GP6) is expressed by platelets as a receptor for the collagen [2]. The platelet membrane GPVI receptor plays an important role in coagulation processes. It has been shown that the GPVI receptor expression is genetically determined and that polymorphisms in these genes may change the expression of GPVI receptor and influence the coagulation processes [3].

*PEAR1* is called aggregation receptor 1, and is expressed on platelets and endothelial cells as a type 1 membrane protein; it plays a role in aggregation-induced signaling. It also undergoes tyrosine phosphorylation after platelet–platelet contact [4]. Previous studies have suggested that polymorphisms in the PEAR1 gene may alter the platelet reactivity and may be associated with the development of thromboembolism and cardiovascular diseases [5].

*TRIP1* to *TRIP15* genes encode thyroid hormone receptor β (TRβ)-binding proteins. Among the 15 *TRIP* genes, the human *TRIP8* gene (also known as *JMJD1C*) consists of 26 exons and is localized on chromosome 10 (10q21.3) [6]. The *JMJD1C* gene encodes a histone demethylase that regulates the synthesis of thyroid hormone and androgen receptors, and it is expressed in pluripotent cells [7,8,9].

IRAG (also known as *MRVI1*, *JEDI*, and *MEGF12*) is an inositol trisphosphate receptor-associated cGMP kinase substrate which is an endoplasmic reticulum-anchored membrane protein. A deficiency of this protein or mutation in the *IRAG* gene can impair smooth muscle relaxation and reduce the inhibition of platelet activation [10,11]. A study with mice proved that IRAG is involved in the blocking of platelet aggregation [12].

The *PIK3CG* gene is located on chromosome 7 and encodes an enzyme that phosphorylates phosphoinositides. PI3Kγ is expressed in cardiomyocytes, endothelial cells, fibroblasts, and vascular smooth muscle cells, and it plays a role in myocardial metabolism [13].

The morphogen sonic hedgehog (SHH) is a protein in humans that is encoded by the *SHH* gene on chromosome 7. Previous studies suggest that polymorphism in the SHH gene may be associated with platelet aggregation [1], and the SHH protein regulates angiogenesis and tissue regeneration by modulating the expression of multiple growth factors [14,15,16,17].

The aim of this study was to examine the associations between polymorphisms in *GP6* (rs1671152), *PEAR1A* (rs12566888), *MRVI1* (rs7940646), *PIK3CG* (rs342286), *JMJD1C* (rs10761741), and *SHH* (rs2363910) and CAD in the form of unstable angina, as well as to study selected clinical and biochemical parameters.

## 2. Materials and Methods

### 2.1. Patients

This study enrolled 246 patients (age 62.7 ± 9.9) with unstable angina that was diagnosed in the years 2017–2018. Unstable angina was diagnosed on the basis of typical clinical symptoms, ST segment or T wave changes in the electrocardiography (ECG), and >70% stenosis of at least one major coronary artery in coronary angiography. The control group consisted of 189 healthy control subjects (age 65.5 ± 11.0). The controls (*n* = 189) were subjects with negative findings following coronary angiography. The patients were recruited in accordance with the principles of the Declaration of Helsinki, and the study was approved by the ethics committee at Pomeranian Medical University (KB-0012/46/17), Szczecin, Poland; written informed consent was obtained from all subjects.

### 2.2. Genotyping

DNA was isolated from peripheral blood using the GenElute Mammalian Genomic DNA Miniprep Kit (Sigma-Aldrich, St. Louis, MO, USA) according to the manufacturer’s protocol. All the samples were genotyped in duplicates using an allelic discrimination assay on a CFX Connect Real-Time PCR Detection System (Bio-Rad, Feldkirchen, Germany) with TaqMan^®^ probes.

### 2.3. Statistical Analysis

The consistency of the genotype distribution with the Hardy–Weinberg equilibrium (HWE) was assessed using the exact test. The genotype and allele distributions were compared between groups using chi-square test; the non-parametric Mann–Whitney U test was used to compare the clinical parameters between groups. *p* < 0.05 was considered statistically significant.

## 3. Results

The distributions of the studied polymorphisms were in HWE. The distribution of the studied polymorphisms in patients with unstable angina and in the control subjects is presented in Table 1 and Table 2. As shown, there were no significant differences in the distribution of the studied polymorphisms between patients with unstable angina and the controls.

Additionally, we examined the associations between the studied polymorphisms and clinical parameters, such as BMI, waist circumference, total cholesterol, HDL, LDL, and triacylglycerols (Table 3, Table 4, Table 5, Table 6, Table 7 and Table 8). We observed increased BMI values in carriers of the *GP6* rs1671152 GG genotype and lower TG levels in carriers of the *SHH* rs2363910 GG genotype.

We also examined the associations between the studied genotypes and the frequency of diagnosis of type 2 diabetes mellitus and hypertension (Table 9 and Table 10). Type 2 diabetes mellitus was more frequently diagnosed in carriers of the *GP6* rs1671152 GG genotype (Table 9). There were no statistically significant associations between the studied polymorphisms and the frequency of hypertension (data not shown).

## 4. Discussion

In this study, we examined the associations between polymorphisms in *GP6* (rs1671152), *PEAR1A* (rs12566888), *MRVI1* (rs7940646), *PIK3CG* (rs342286), *JMJD1C* (rs10761741), and *SHH* (rs2363910) and unstable angina. Our results suggest a lack of statistically significant associations. Our results showed that type 2 diabetes mellitus was more frequently diagnosed in carriers of the *GPVI* rs1671152 GG genotype. Moreover, patients with this genotype had higher BMI values.

Previous studies have indicated that platelet GPVI (GP6) plays an important role in platelet function and hyperglycemia, and diabetes may alter the expression of GPVI [18]. Studies have suggested that patients with type 2 diabetes have an increased platelet expression of GPVI, which correlates with atherothrombotic complications and the frequency of cardiovascular events in these patients [19]. Additionally, patients with acute ischemic stroke had increased plasma levels of GPVI [20].

Previous studies have suggested that the SNPs in the *GPVI* gene may modulate the platelet reactivity. Postula et al. [21] investigated the effect of 27 SNPs (also, rs342286 in *PIK3CG*, rs1671152 in *GPVI*, and rs12566888 in *PEAR1*) on the platelet reactivity in 304 Caucasian diabetic patients and indicated a significance for two SNPs in the *GPVI* gene: rs1671152 and rs1613662. The authors recognized that the differences in genes associated with platelet receptors may alter the platelet reactivity and may change the efficacy of platelet inhibitors [22]. In another 12-year prospective study [23], the authors evaluated the variability of the *GPVI* gene in a group of patients with sticky platelet syndrome manifested as miscarriage. The authors determined the relationship between 15 selected SNPs of the *GP6* gene in the studied syndrome compared to the control participants. The haplotype analysis showed a significantly higher occurrence of haplotypes combined with rs1671152, rs2304167, rs1654416, and rs1613662 in the *GPVI* gene, which results in a variant of the GPVI protein, resulting in a 4.5-fold increase in the risk of fetal loss in patients versus healthy controls. Some authors [24,25] have suggested that the mechanism involved in the defected action of GPVI has a significant effect on GPVI-mediated signal transduction through tyrosine phosphorylation in a protein named Syk. The 10-fold increase in the risk of myocardial infarction associated with the possession of the 13254CC genotype was reported in a study with 525 MI patients and 474 healthy control subjects from the UK. Only this polymorphism was selected in the study because it contributes to the resulting substitution of serine 219 by proline and influences GPVI function [26]. It would be important to analyze the *GPVI* gene polymorphism in patients with only diabetes.

The increased expression of GPVI plays an important role in platelet hyperactivity in patients with cardiovascular diseases [20,27]. It has been shown that hyperglycemia and diabetes may alter the expression of GPVI [19]. Previous studies suggest that patients with diabetes have elevated levels of platelet GPVI and ROS generation [28]. The combination of high glucose levels, increased oxidative stress, elevated platelet GPVI, and platelet reactivity in patients with diabetes makes this group of patients especially exposed to the development of cardiovascular diseases. Arthur et al. indicated that hyperglycemia in monkey with experimental diabetes induces ROS generation with the engagement of GPVI. The inhibition of GPVI significantly reduced the ROS generation in diabetic monkeys [29]. These results indicate that glycemic control plays a crucial role in reducing GPVI-dependent platelet hyperreactivity.

The expression of GPVI on platelets is also altered in patients with diseases of the circulatory system [30,31,32]. GPVI plays an important role in the activation of circulating platelets in acute coronary syndrome, ischemic stroke, and diabetes mellitus [30,31,32]. The studies suggest that elevated GPVI levels may be the marker of acute coronary syndromes and ischemic stroke [30,31,32].

Moreover, recent studies have indicated the clinical significance of GPVI in sepsis and cancer metastasis.

Soluble GPVI is a marker of platelet activation in thrombotic conditions. Montague et al. suggested that soluble GPVI is an important platelet-specific marker for platelet activation that predicts sepsis progression and mortality in injured patients [33]. Mammadova-Bach et al. have shown that GPVI plays an important role in cancer metastasis [34]. The genetic deficiency of platelet GPVI in mice decreased the experimental and spontaneous metastasis of colon and breast cancer cells. These authors suggest that GPVI may be a promising target for antimetastatic therapies.

Several studies have examined the associations between polymorphisms in genes related to platelet reactivity with the risk of CAD. As described, the *JMJD1C* gene variant (rs7896518), located in an intron, showed an association with the platelet count and volume in European [35] and African American populations [35]. On the other hand, while some showed that rs10761741 in the *JMJD1C* gene was associated with epinephrine-induced platelet aggregation and with higher circulating VEGF levels in a European population [36,37], others showed that the same variant in the *JMJD1C* gene was not associated with higher circulating VEGF levels [38]. It is important to know the roles of platelets and VEGF in the development of atherosclerosis and arterial thrombosis [38].

The results of the meta-analysis by Johnson et al. suggest that polymorphisms in the genes *PEAR1* (rs12566888), *MRVI1* (rs7940646), *GPVI* (rs1671152), and *SHH* (rs2363910) may be associated with differences in platelet aggregation [1]. Epinephrine-induced platelet aggregation was associated with SNPs in *PEAR1* (rs12566888), *PIK3CG* (rs342286), and *JMJD1C* (rs10761741) [39]. In a genome-wide association study of 3000 unrelated men of European origin, the authors identified *MRVI1* (rs7940646) as a gene variant associated with platelet reactivity, *PIK3CG* (rs342286) as a gene variant associated with increased mean platelet volume, *JMJD1C* (rs10761741) as a gene variant associated with decreased platelet reactivity, and *PEAR1* (rs12566888) as a gene variant associated with reduced ADP-induced platelet aggregation [40].

As we have already reported, the rs342286 variant of the *PIK3CG* gene is associated with a younger age of patients with acute coronary syndrome [41]. In a study by Appelboom et al. on patients after intracerebral hemorrhage, there was a positive association between rs342286 and hematoma volume in the prospective assessment of computed tomography scans [42].

## 5. Conclusions

The results of this study suggest a lack of association between *GP6* (rs1671152), *PEAR1A* (rs12566888), *MRVI1* (rs7940646), *PIK3CG* (rs342286), *JMJD1C* (rs10761741), *SHH* (rs2363910), and unstable angina. The results indicate a positive association between the GG genotype of the *GP6* (rs1671152) polymorphism and type 2 diabetes.

## Figures and Tables

**Table 1:** Distributions of the *PEAR1A*, *MRVI1*, and *GP6* genotypes and alleles in patients with unstable angina and controls.

	Control Group		Unstable Angina		*p* Value ^^^		*p* Value ^^^	OR (95% CI)
	*n*	%	*n*	%				
***PEAR1A*** **rs12566888** **Genotype**								
GG	152	80.85%	199	83.97%	0.57	TT+GT vs. GG	0.40	0.81 (0.49–1.33)
GT	34	18.09%	37	15.61%		TT vs. GT+GG	0.43	0.39 (0.04–4.38)
TT	2	1.06%	1	0.42%		TT vs. GG	0.42	0.38 (0.03–4.25)
						GT vs. GG	0.48	0.83 (0.50–1.39)
						TT vs. GT	0.52	0.46 (0.04–5.30)
**Allele**								
G	338	89.89%	435	91.77%		T vs. G	0.34	0.80 (0.50–1.27)
T	38	10.11%	39	8.23%				
***MRVI1*** **rs7940646** **genotype**								
CC	80	42.33%	100	40.65%	0.82	TT+CT vs. CC	0.72	1.07 (0.73–1.57)
CT	86	45.50%	119	48.37%		TT vs. CT+CC	0.70	0.89 (0.49–1.61)
TT	23	12.17%	27	10.98%		TT vs. CC	0.84	0.94 (0.50–1.76)
						CT vs. CC	0.62	1.11 (0.74–1.66)
						TT vs. CT	0.60	0.85 (0.46–1.58)
**Allele**								
C	246	65.08%	319	64.84%		T vs. C	0.94	1.01 (0.76–1.34)
T	132	34.92%	173	35.16%				
***GP6*** **rs1671152** **genotype**								
GG	157	83.07%	206	83.74%	0.49	TT+GT vs. GG	0.85	0.95 (0.57–1.59)
GT	30	15.87%	34	13.82%		TT vs. GT+GG	0.29	2.34 (0.47–11.71)
TT	2	1.06%	6	2.44%		TT vs. GG	0.30	2.29 (0.46–11.48)
						GT vs. GG	0.59	0.86 (0.51–1.47)
						TT vs. GT	0.24	2.65 (0.50–14.12)
**Allele**								
G	344	91.01%	446	90.65%		T vs. G	0.86	1.04 (0.66–1.66)
T	34	8.99%	46	9.35%				

^ χ^2^ test; Alleles: A–adenine, C–cytosine, G–guanine, T–thymine; HWE: control group *p* = 1.00, unstable angina *p* = 1.00 for *PEAR1A* rs12566888; HWE: control group *p* = 1.00, unstable angina *p* = 0.40 for *MRVI1* rs7940646; HWE: control group *p* = 0.65, unstable angina *p* = 0.011 for *GP6* rs1671152.

**Table 2:** Distributions of the *PIK3CG*, *JMJD1C*, and *SHH* genotypes and alleles in patients with unstable angina and controls.

	Control Group		Unstable Angina		*p* Value ^^^		*p* Value ^^^	OR (95% CI)
	*n*	%	*n*	%				
***PIK3CG* rs342286** **genotype**								
AA	55	29.26%	65	26.75%	0.76	GG+AG vs. AA	0.56	1.13 (0.74–1.73)
AG	98	52.13%	127	52.26%		GG vs. AG+AA	0.54	1.16 (0.72–1.88)
GG	35	18.62%	51	20.99%		GG vs. AA	0.46	1.23 (0.70–2.16)
						AG vs. AA	0.69	1.10 (0.70–1.71)
						GG vs. AG	0.65	1.12 (0.68–1.86)
**Allele**								
A	208	55.32%	257	52.88%		G vs. A	0.48	1.10 (0.84–1.45)
G	168	44.68%	229	47.12%				
***JMJD1C* rs10761741** **genotype**								
GG	68	36.36%	86	35.10%	0.70	TT+GT vs. GG	0.79	1.06 (0.71–1.57)
GT	89	47.59%	112	45.71%		TT vs. GT+GG	0.40	1.24 (0.75–2.06)
TT	30	16.04%	47	19.18%		TT vs. GG	0.45	1.24 (0.71–2.16)
						GT vs. GG	0.98	1.00 (0.65–1.52)
						TT vs. GT	0.42	1.24 (0.73–2.13)
**Allele**								
G	225	60.16%	284	57.96%		T vs. G	0.51	1.10 (0.83–1.44)
T	149	39.84%	206	42.04%				
***SHH*** **rs2363910** **genotype**								
GG	155	82.01%	199	81.22%	0.93	TT+GT vs. GG	0.83	1.05 (0.65–1.72)
GT	33	17.46%	44	17.96%		TT vs. GT+GG	0.72	1.55 (0.14–17.19)
TT	1	0.53%	2	0.82%		TT vs. GG	0.72	1.56 (0.14–17.34)
						GT vs. GG	0.88	1.04 (0.63–1.71)
						TT vs. GT	0.74	1.50 (0.13–17.25)
**Allele**								
G	343	90.74%	442	90.20%		T vs. G	0.79	1.06 (0.67–1.68)
T	35	9.26%	48	9.80%				

^ χ^2^ test; HWE: control group *p* = 0.55, unstable angina *p* = 0.52 for *PIK3CG* rs342286; HWE: control group *p* = 1.00, unstable angina *p* = 0.36 for *JMJD1C* rs10761741; HWE: control group *p* = 1.00, unstable angina *p* = 1.00 for *SHH* rs2363910.

**Table 3:** Associations between the clinical parameters of patients with unstable angina and the *PEAR1A* rs12566888 genotypes.

Parameters	*PEAR1A* rs12566888 Genotype								
		GG		GT		GG+GT	*n*	GT+TT	GG vs. GT+TT
	*n*	Mean ± SD	*n*	Mean ± SD	*n*	Mean ± SD	*n*	Mean ± SD	p ^&^
Age [years]	199	62.8 ± 10.0	37	62.0 ± 8.5	236	62.6 ± 9.8	38	62.0 ± 8.4	0.64
BMI [kg/m^2^]	199	28.2 ± 3.9	37	28.6 ± 3.8	236	28.3 ± 3.8	38	28.5 ± 3.7	0.78
Waist [cm]	198	95.2 ± 10.0	37	96.5 ± 10.1	235	95.4 ± 10.0	38	96.4 ± 10.0	0.54
CH [mg/dL]	180	237.4 ± 63.0	33	228.1 ± 56.9	213	236.0 ± 62.1	34	228.6 ± 56.1	0.56
HDL [mg/dL]	144	45.4 ± 8.4	32	44.6 ± 8.7	176	45.3 ± 8.5	33	44.6 ± 8.6	0.52
LDL [mg/dL]	144	169.6 ± 57.7	32	162.2 ± 55.3	176	168.3 ± 57.1	33	162.5 ± 54.4	0.48
TG [mg/dL]	173	144.7 ± 74.3	33	149.4 ± 99.1	206	145.5 ± 78.6	34	149.3 ± 97.6	0.88

^&^—Mann–Whitney U test; BMI—body mass index; CH—total cholesterol in serum; HDL—high density cholesterol in serum; LDL—low density cholesterol in serum; TG—triacylglycerols in serum.

**Table 4:** Associations between the clinical parameters of patients with unstable angina and the *MRVI1* rs7940646 genotypes.

Parameters	*MRVI1* rs7940646 Genotype												
		CC		CT		TT		CC+CT	*n*	CT+TT	CC vs. CT+TT	CC+CT vs. TT	CC vs. TT
	*n*	Mean ± SD	*n*	Mean ± SD	*n*	Mean ± SD	*n*	Mean ± SD	*n*	Mean ± SD	p ^&^		
Age [years]	100	63.3 ± 9.8	119	62.0 ± 10.2	27	63.0 ± 9.0	219	62.6 ± 10.0	146	62.2 ± 9.9	0.43	0.78	0.94
BMI [kg/m^2^]	100	28.5 ± 4.4	119	28.4 ± 3.5	27	27.7 ± 3.1	219	28.4 ± 3.9	146	28.3 ± 3.4	0.78	0.39	0.43
Waist [cm]	99	95.6 ± 10.7	119	95.4 ± 9.8	27	97.0 ± 11.2	218	95.5 ± 10.2	146	95.7 ± 10.1	0.72	0.70	0.66
CH [mg/dL]	92	229.9 ± 54.9	108	239.1 ± 66.0	23	245.7 ± 69.9	200	234.9 ± 61.2	131	240.3 ± 66.5	0.37	0.43	0.33
HDL [mg/dL]	80	45.1 ± 8.7	86	45.6 ± 8.1	17	45.0 ± 9.3	166	45.3 ± 8.3	103	45.5 ± 8.2	0.79	0.94	0.98
LDL [mg/dL]	80	158.8 ± 47.8	86	174.3 ± 59.3	17	186.2 ± 77.4	166	166.8 ± 54.5	103	176.3 ± 62.4	0.079	0.41	0.19
TG [mg/dL]	91	143.5 ± 79.0	103	148.9 ± 79.1	22	136.3 ± 61.2	194	146.4 ± 78.9	125	146.7 ± 76.2	0.69	0.69	0.84

^&^—Mann–Whitney U test; BMI—body mass index; CH—total cholesterol in serum; HDL—high density cholesterol in serum; LDL—low density cholesterol in serum; TG—triacylglycerols in serum.

**Table 5:** Associations between the clinical parameters of patients with unstable angina and the *GP6* rs1671152 genotypes.

Parameters	*GP6* rs1671152 Genotype												
		GG		GT		TT		GG+GT	*n*	GT+TT	GG vs. GT+TT	GG+GT vs. TT	GG vs. TT
	*n*	Mean ± SD	*n*	Mean ± SD	*n*	Mean ± SD	*n*	Mean ± SD	*n*	Mean ± SD	p ^&^		
Age [years]	206	62.9 ± 9.9	34	61.1 ± 10.0	6	63.7 ± 8.2	240	62.6 ± 9.9	40	61.5 ± 9.7	0.32	0.79	0.85
BMI [kg/m^2^]	206	28.6 ± 3.8	34	27.2 ± 3.7	6	26.3 ± 4.1	240	28.4 ± 3.8	40	27.1 ± 3.8	0.021	0.13	0.10
Waist [cm]	206	96.0 ± 10.3	33	94.1 ± 10.7	6	91.2 ± 11.0	239	95.8 ± 10.3	39	93.6 ± 10.7	0.21	0.21	0.19
CH [mg/dL]	187	234.9 ± 62.5	30	241.3 ± 62.7	6	246.0 ± 50.4	217	235.7 ± 62.4	36	242.1 ± 60.2	0.34	0.43	0.41
HDL [mg/dL]	153	45.3 ± 8.5	25	45.3 ± 8.2	5	45.8 ± 6.0	178	45.3 ± 8.5	30	45.4 ± 7.7	0.80	0.73	0.71
LDL [mg/dL]	153	167.1 ± 57.8	25	173.1 ± 55.8	5	193.4 ± 38.0	178	167.9 ± 57.4	30	176.5 ± 53.2	0.21	0.19	0.18
TG [mg/dL]	181	145.2 ± 77.9	29	154.2 ± 77.2	6	107.5 ± 45.5	210	146.4 ± 77.7	35	146.2 ± 74.4	0.93	0.17	0.18

^&^—Mann–Whitney U test; BMI—body mass index; CH—total cholesterol in serum; HDL—high density cholesterol in serum; LDL—low density cholesterol in serum; TG—triacylglycerols in serum.

**Table 6:** Associations between the clinical parameters of patients with unstable angina and the *PIK3CG* genotypes.

Parameters	*PIK3CG* rs342286 Genotype												
		AA		AG		GG		AA+AG	*n*	AG+GG	AA vs. AG+GG	AA+AG vs. GG	AA vs. GG
	*n*	Mean ± SD	*n*	Mean ± SD	*n*	Mean ± SD	*n*	Mean ± SD	*n*	Mean ± SD	p ^&^		
Age [years]	65	64.2 ± 9.1	127	62.5 ± 10.0	51	61.1 ± 10.5	192	63.1 ± 9.7	178	62.1 ± 10.2	0.16	0.19	0.091
BMI [kg/m^2^]	65	28.0 ± 3.4	127	28.5 ± 3.9	51	28.1 ± 4.4	192	28.4 ± 3.7	178	28.4 ± 4.0	0.73	0.57	0.83
Waist [cm]	64	95.6 ± 9.7	127	95.5 ± 10.7	51	95.7 ± 10.5	191	95.5 ± 10.3	178	95.5 ± 10.6	0.97	0.84	0.83
CH [mg/dL]	60	238.8 ± 70.6	113	234.0 ± 59.4	48	237.3 ± 58.8	173	235.6 ± 63.3	161	234.9 ± 59.1	0.84	0.59	0.55
HDL [mg/dL]	45	47.7 ± 8.2	94	44.4 ± 8.7	42	44.5 ± 7.6	139	45.5 ± 8.7	136	44.4 ± 8.4	0.027	0.67	0.11
LDL [mg/dL]	45	172.6 ± 60.4	94	167.6 ± 57.4	42	167.0 ± 50.0	139	169.2 ± 59.4	136	167.4 ± 55.0	0.84	0.99	0.94
TG [mg/dL]	58	143.2 ± 74.2	110	148.8 ± 87.0	46	139.1 ± 55.3	168	146.9 ± 82.6	156	145.9 ± 78.9	0.70	0.82	0.69

^&^—Mann–Whitney U test; BMI—body mass index; CH—total cholesterol in serum; HDL—high density cholesterol in serum; LDL—low density cholesterol in serum; TG—triacylglycerols in serum.

**Table 7:** Associations between the clinical parameters of patients with unstable angina and the *JMJD1C* rs10761741 genotypes.

Parameters	*JMJD1C* rs10761741 Genotype												
		GG		GT		TT		GG+GT	*n*	GT+TT	GG vs. GT+TT	GG+GT vs. TT	GG vs. TT
	*n*	Mean ± SD	*n*	Mean ± SD	*n*	Mean ± SD	*n*	Mean ± SD	*n*	Mean ± SD	p ^&^		
Age [years]	86	63.7 ± 9.5	112	62.4 ± 10.4	47	61.5 ± 9.4	198	63.0 ± 10.0	159	62.1 ± 10.1	0.20	0.31	0.15
BMI [kg/m^2^]	86	28.2 ± 3.6	112	28.6 ± 4.2	47	27.9 ± 3.6	198	28.4 ± 3.9	159	28.4 ± 4.0	0.74	0.39	0.62
Waist [cm]	85	95.6 ± 10.2	112	95.8 ± 11.0	47	95.2 ± 9.0	197	95.7 ± 10.6	159	95.6 ± 10.4	0.94	0.61	0.72
CH [mg/dL]	80	243.0 ± 63.7	96	230.4 ± 54.9	46	236.8 ± 72.9	176	236.1 ± 59.2	142	232.4 ± 61.1	0.15	0.55	0.27
HDL [mg/dL]	69	45.5 ± 8.4	79	45.7 ± 7.9	35	44.1 ± 9.5	148	45.6 ± 8.1	114	45.2 ± 8.4	0.74	0.22	0.33
LDL [mg/dL]	69	174.2 ± 60.9	79	161.9 ± 51.4	35	172.9 ± 60.9	148	167.6 ± 56.2	114	165.2 ± 54.5	0.37	0.89	0.69
TG [mg/dL]	77	158.8 ± 96.8	94	135.4 ± 59.0	44	144.8 ± 70.9	171	146.0 ± 78.9	138	138.4 ± 62.9	0.18	1.00	0.52

^&^—Mann–Whitney U test; BMI—body mass index; CH—total cholesterol in serum; HDL—high density cholesterol in serum; LDL—low density cholesterol in serum; TG—triacylglycerols in serum.

**Table 8:** Associations between the clinical parameters of patients with unstable angina and the *SHH* rs2363910 genotypes.

Parameters	*SHH* rs2363910 Genotype												
		GG		GT		TT		GG+GT	*n*	GT+TT	GG vs. GT+TT	GG+GT vs. TT	GG vs. TT
	*n*	Mean ± SD	*n*	Mean ± SD	*n*	Mean ± SD	*n*	Mean ± SD	*n*	Mean ± SD	p ^&^		
Age [years]	199	61.9 ± 9.8	44	66.1 ± 9.6	2	59.0 ± 17.0	243	62.7 ± 9.9	46	65.8 ± 9.8	0.029	0.62	0.66
BMI [kg/m^2^]	199	28.4 ± 3.8	44	27.9 ± 3.8	2	29.0 ± 2.8	243	28.3 ± 3.8	46	28.0 ± 3.8	0.40	0.67	0.71
Waist [cm]	198	95.3 ± 10.0	44	97.0 ± 12.0	2	98.0 ± 2.8	242	95.6 ± 10.4	46	97.1 ± 11.7	0.54	0.76	0.74
CH [mg/dL]	180	233.9 ± 59.4	40	245.0 ± 73.3	2	271.5 ± 68.6	220	235.9 ± 62.1	42	246.2 ± 72.5	0.42	0.34	0.31
HDL [mg/dL]	154	45.4 ± 8.5	27	44.5 ± 8.1	2	45.5 ± 3.5	181	45.3 ± 8.4	29	44.6 ± 7.8	0.75	0.80	0.81
LDL [mg/dL]	154	164.7 ± 53.4	27	189.8 ± 73.1	2	182.5 ± 40.3	181	168.5 ± 57.2	29	189.3 ± 70.9	0.090	0.49	0.44
TG [mg/dL]	176	139.1 ± 70.5	37	172.2 ± 102.1	2	182.0 ± 39.6	213	144.9 ± 77.6	39	172.7 ± 99.6	0.036	0.21	0.19

^&^—Mann–Whitney U test; BMI—body mass index; CH—total cholesterol in serum; HDL—high density cholesterol in serum; LDL—low density cholesterol in serum; TG—triacylglycerols in serum.

**Table 9:** Distributions of the *PEAR1A*, *MRVI1*, and *GP6* genotypes and alleles in unstable angina patients with and without diabetes mellitus (DM).

	Without Diabetes Mellitus		Diabetes Mellitus		*p* Value ^^^		*p* Value ^^^	OR (95% CI)
	*n*	%	*n*	%				
***PEAR1A* rs12566888** **genotype**								
GG	149	82.32%	50	89.29%	0.43	TT+GT vs. GG	0.21	0.56 (0.22–1.41)
GT	31	17.13%	6	10.71%		TT vs. GT+GG	0.58	0 (-)
TT	1	0.55%	0	0.00%		TT vs. GG	0.56	0 (-)
						GT vs. GG	0.24	0.58 (0.23–1.46)
						TT vs. GT	0.66	0 (-)
**Allele**								
G	329	90.88%	106	94.64%		T vs. G	0.21	0.56 (0.23–1.38)
T	33	9.12%	6	5.36%				
***MRVI1*** **rs7940646** **genotype**								
CC	78	41.94%	22	36.67%	0.46	TT+CT vs. CC	0.47	1.25 (0.68–2.27)
CT	86	46.24%	33	55.00%		TT vs. CT+CC	0.45	0.68 (0.24–1.88)
TT	22	11.83%	5	8.33%		TT vs. CC	0.69	0.81 (0.27–2.37)
						CT vs. CC	0.33	1.36 (0.73–2.53)
						TT vs. CT	0.32	0.59 (0.21–1.69)
**Allele**								
C	242	65.05%	77	64.17%		T vs. C	0.86	1.04 (0.68–1.60)
T	130	34.95%	43	35.83%				
***GP6*** **rs1671152** **genotype**								
GG	149	80.11%	57	95.00%	0.022	TT+GT vs. GG	0.007	0.21 (0.06–0.71)
GT	31	16.67%	3	5.00%		TT vs. GT+GG	0.16	0 (-)
TT	6	3.23%	0	0.00%		TT vs. GG	0.13	0 (-)
						GT vs. GG	0.019	0.25 (0.07–0.86)
						TT vs. GT	0.45	0 (-)
**Allele**								
G	329	88.44%	117	97.50%		T vs. G	0.0030	0.20 (0.06–0.64)
T	43	11.56%	3	2.50%				

^ χ^2^ test.

**Table 10:** Distributions of the *PIK3CG*, *JMJD1C*, and *SHH* genotypes and alleles in unstable angina patients with and without diabetes mellitus (DM).

	Without Diabetes Mellitus		Diabetes Mellitus		*p* Value ^^^		*p* Value ^^^	OR (95% CI)
	n	%	n	%				
***PIK3CG*** **rs342286** **genotype**								
AA	47	25.54%	18	30.51%	0.75	GG+AG vs. AA	0.45	0.78 (0.41–1.49)
AG	98	53.26%	29	49.15%		GG vs. AG+AA	0.89	0.95 (0.46–1.96)
GG	39	21.20%	12	20.34%		GG vs. AA	0.61	0.80 (0.35–1.87)
						AG vs. AA	0.46	0.77 (0.39–1.53)
						GG vs. AG	0.92	1.04 (0.48–2.24)
**Allele**								
A	192	52.17%	65	55.08%		G vs. A	0.58	0.89 (0.59–1.35)
G	176	47.83%	53	44.92%				
***JMJD1C*** **rs10761741** **genotype**								
GG	66	35.48%	20	33.90%	0.59	TT+GT vs. GG	0.82	1.07 (0.58–1.99)
GT	87	46.77%	25	42.37%		TT vs. GT+GG	0.31	1.44 (0.71–2.93)
TT	33	17.74%	14	23.73%		TT vs. GG	0.41	1.40 (0.63–3.12)
						GT vs. GG	0.88	0.95 (0.49–1.85)
						TT vs. GT	0.32	1.48 (0.69–3.18)
**Allele**								
G	219	58.87%	65	55.08%		T vs. G	0.47	1.17 (0.77–1.77)
T	153	41.13%	53	44.92%				
***SHH*** **rs2363910** **genotype**								
GG	153	82.26%	46	77.97%	0.48	TT+GT vs. GG	0.46	1.31 (0.64–2.70)
GT	31	16.67%	13	22.03%		TT vs. GT+GG	0.42	-
TT	2	1.08%	0	0.00%		TT vs. GG	0.44	-
						GT vs. GG	0.37	1.39 (0.67–2.88)
						TT vs. GT	0.36	-
**Allele**								
G	337	90.59%	105	88.98%		T vs. G	0.61	1.19 (0.61–2.34)
T	35	9.41%	13	11.02%				

^ χ^2^ test.
